# Draft Genome Sequence of a Fermenting Bacterium, *Soehngenia* sp. Strain 1933P, Isolated from a Petroleum Reservoir in Azerbaijan

**DOI:** 10.1128/MRA.00689-19

**Published:** 2019-07-18

**Authors:** Denis S. Grouzdev, Salimat K. Bidzhieva, Diyana S. Sokolova, Tatiyana P. Tourova, Andrey B. Poltaraus, Tamara N. Nazina

**Affiliations:** a Institute of Bioengineering, Research Center of Biotechnology, Russian Academy of Sciences, Moscow, Russian Federation; b Winogradsky Institute of Microbiology, Research Center of Biotechnology, Russian Academy of Sciences, Moscow, Russian Federation; c Engelhardt Institute of Molecular Biology, Russian Academy of Sciences, Moscow, Russian Federation; Indiana University, Bloomington

## Abstract

The draft genome sequence of a mesophilic fermenting bacterium, Soehngenia sp. strain 1933P, isolated from production water of the Binagady petroleum reservoir (Republic of Azerbaijan), is presented. The genome is annotated for elucidation of the metabolic potential and taxonomic position of strain 1933P.

## ANNOUNCEMENT

The mesophilic fermenting bacterium Soehngenia sp. strain 1933P (VKM B-3382) was isolated from a methanogenic enrichment culture obtained from the Binagady petroleum reservoir (Republic of Azerbaijan) (1). The methanogenic enrichment was cultivated in the mineral medium with methanol (5% [vol/vol]) and 500 mg Na_2_S · 9H_2_O (2) and contained fermentative bacteria and methanogens of the genus Methanosarcina (3). Several flasks with the methanogenic enrichment were stored at room temperature (18 to 24°C) for 33 years without transfers to fresh medium for studying the survival of oilfield communities. Then, the enrichment was transferred to fresh medium with methanol, and a pure culture of strain 1933P was isolated by sequential transfers from the highest dilutions on the mineral medium (2) with peptone (2 g liter^−1^), yeast extract (0.2 g liter^−1^), 1.5% NaCl, and Na_2_S · 9H_2_O (0.2 g · liter^−1^) at 30°C. To ascertain the culture purity, material from the highest growth-positive dilution (10^−7^) was used to inoculate the fresh medium with peptone. After 5 to 7 days of incubation at 30°C, the 16S rRNA gene was amplified with the 27F and 1492R primers (4), and purified PCR products were sequenced with an ABI Prism 3730 DNA analyzer (Applied Biosystems, USA). The 16S rRNA sequence analysis using a BLASTn (5) search against the NCBI database revealed that strain 1933P was phylogenetically closely related (98.5% similarity) to Soehngenia saccharolytica DSM 12858^T^ (6), a strain from the only species of the genus Soehngenia. The aim of the present study was to sequence the genome of the strain 1933P in order to elucidate its metabolic potential and taxonomic position.

Strain 1933P was grown anaerobically at 30°C in the mineral medium (2) used for the isolation of pure culture. Cells were harvested from 2 liters of culture medium by centrifugation after 7 days of incubation. DNA was purified from the cell biomass using the cetyltrimethylammonium bromide (CTAB) method (7). The libraries were constructed with the NEBNext DNA library prep reagent set for Illumina, according to the protocol for the kit. Sequencing of genomic DNA was carried out using the HiSeq 2500 platform (Illumina, Inc., USA), with 150-bp paired-end reads. A total of 4,678,392 reads were obtained from strain 1933P. Raw sequence reads were quality checked with FastQC version 11.7 (https://www.bioinformatics.babraham.ac.uk/projects/fastqc/), and low-quality reads were trimmed using Trimmomatic version 0.36 (8), with the default settings for paired-end reads. Subsequently, the quality-filtered reads were *de novo* assembled with SPAdes version 3.13.0 using the default settings (9). The final assembled 1,917,091-bp-long genome comprised 33 scaffolds, with an *N*_50_ value of 132,646 bp, G+C content of 31.9%, and coverage of 630×. The average nucleotide identity (ANI) (10) and digital DNA-DNA hybridization (dDDH) (http://ggdc.dsmz.de/ggdc.php) (11) values of 83.5% and 27.0%, respectively, to the genome of the most closely related species, S. saccharolytica DSM 12858^T^, were below the species cutoffs (95 to 96% for ANI and 70% for dDDH) (12), which indicates that strain 1933P belongs to a new *Soehngenia* species.

Identification of protein-coding sequences and primary annotation were performed using the NCBI Prokaryotic Genome Automatic Annotation Pipeline (PGAAP) (13). The draft genome sequence of *Soehngenia* sp. 1933P contained 1,853 genes, of which 1,789 were protein-coding sequences, 23 were pseudogenes, and 41 coded for RNAs. Functional annotation of the genome performed with the Rapid Annotations using Subsystems Technology (RAST) server (14, 15), via the RAST*tk* pipeline with the default settings (16), revealed that 150 of the genes were associated with protein metabolism, 106 genes were associated with the metabolism of amino acids and derivatives, 101 genes were associated with carbohydrate metabolism, and 40 genes were associated with the metabolism of cofactors, vitamins, prosthetic groups, and pigments. The genome sequence of *Soehngenia* sp. 1933P provided here will broaden the knowledge of the genus *Soehngenia* and the putative importance of its members in subsurface communities.

### Data availability.

The GenBank/EMBL/DDBJ accession number for the 16S rRNA gene sequence of *Soehngenia* sp. strain 1933P is MK712484. This whole-genome shotgun project has been deposited at DDBJ/ENA/GenBank under the accession number SRIB00000000. The version described in this paper is version SRIB01000000. The associated BioProject, BioSample, and SRA accession numbers are PRJNA529817, SAMN11286712, and SRR8846858, respectively. RAST annotation of *Soehngenia* sp. 1933P is publicly available using the guest account for the RAST online database (job ID 707512).
